# Using pharmacists to improve risk stratification and management of stage 3A chronic kidney disease: a feasibility study

**DOI:** 10.1186/s12882-016-0383-7

**Published:** 2016-11-08

**Authors:** Alex R. Chang, Michael Evans, Christina Yule, Larissa Bohn, Amanda Young, Meredith Lewis, Elisabeth Graboski, Bethany Gerdy, William Ehmann, Jonathan Brady, Leah Lawrence, Natacha Antunes, Jamie Green, Susan Snyder, H. Lester Kirchner, Morgan Grams, Robert Perkins

**Affiliations:** 1 Division of Nephrology, Geisinger Health System, 100 N Academy Ave, Danville, PA 17821 USA; 2 Kidney Health Research Institute, Geisinger Health System, 100 N Academy Ave, Danville, PA 17821 USA; 3 Geisinger Health System, Enterprise Pharmacy, Danville, PA USA; 4 Geisinger Health System, Center for Health Research, Danville, PA USA; 5 Geisinger Health System, Health Economics Research and Evaluation, Danville, PA USA; 6 Geisinger Health System, Biomedical and Translational Informatics, Danville, PA USA; 7 Johns Hopkins University, Welch Center for Prevention, Epidemiology and Clinical, Baltimore, MD USA; 8 Bayer HealthCare, Whippany, NJ USA

**Keywords:** Pharmacist medication therapy management, Chronic kidney disease, Albuminuria, Proteinuria, Screening, KDIGO guidelines

## Abstract

**Background:**

Measurement of albuminuria to stratify risk in chronic kidney disease (CKD) is not done universally in the primary care setting despite recommendation in KDIGO (Kidney Disease Improving Global Outcomes) guidelines. Pharmacist medication therapy management (MTM) may be helpful in improving CKD risk stratification and management.

**Methods:**

We conducted a pragmatic, cluster-randomized trial using seven primary care clinic sites in the Geisinger Health System to evaluate the feasibility of pharmacist MTM in patients with estimated glomerular filtration rate (eGFR) 45–59 ml/min/1.73 m^2^ and uncontrolled blood pressure (≥150/85 mmHg). In the three pharmacist MTM sites, pharmacists were instructed to follow a protocol aimed to improve adherence to KDIGO guidelines on testing for proteinuria and lipids, and statin and blood pressure medical therapy. In the four control clinics, patients received usual care. The primary outcome was proteinuria screening over a follow-up of 1 year. A telephone survey was administered to physicians, pharmacists, and patients in the pharmacist MTM arm at the end of the trial.

**Results:**

Baseline characteristics were similar between pharmacist MTM (*n* = 24) and control (*n* = 23) patients, although pharmacist MTM patients tended to be younger (64 vs. 71 y; *p* = 0.06) and less likely to have diabetes (17 % vs. 35 %; *p* = 0.2) or baseline proteinuria screening (41.7 % vs. 60.9 %, *p* = 0.2). Mean eGFR was 54 ml/min/1.73 m^2^ in both groups. The pharmacist MTM intervention did not significantly improve total proteinuria screening at the population level (OR 2.6, 95 % CI: 0.5–14.0; *p* = 0.3). However, it tended to increase screening of previously unscreened patients (78.6 % in the pharmacist MTM group compared to 33.3 % in the control group; OR 7.3, 95 % CI: 0.96–56.3; *p* = 0.05). In general, the intervention was well-received by patients, pharmacists, and providers, who agreed that pharmacists could play an important role in CKD management. A few patients contacted the research team to express anxiety about having a CKD diagnosis without prior knowledge.

**Conclusions:**

Pharmacist MTM may be useful in improving risk stratification and management of CKD in the primary care setting, although implementation requires ongoing education and multidisciplinary collaboration and careful communication regarding CKD diagnosis. Future studies are needed to establish the effectiveness of pharmacist MTM on slowing CKD progression and improvement in cardiovascular outcomes.

**Trial registration:**

ClinicalTrials.gov, NCT02208674

Registered August 1, 2014, first patient enrolled September 30, 2014

## Background

Chronic kidney disease (CKD) affects one in seven adults in the U.S. and is estimated to account for more than 20 % of annual Medicare expenditures [1, 2]. Optimal screening and treatment strategies for CKD have been recommended by KDIGO (Kidney Disease: Improving Global Outcomes). For example, guidelines recommend using both estimated glomerular filtration rate (eGFR) and quantification of albuminuria (or proteinuria) to stratify renal and cardiovascular risk in CKD patients [3–5]. For patients with non-proteinuric CKD, KDIGO guidelines recommend treatment to a blood pressure goal of ≤140/90. For patients with proteinuric CKD, KDIGO guidelines recommend a lower blood pressure goal of ≤130/80,and the use of angiotensin converting enzyme inhibitors (ACEIs) or angiotensin receptor blockers (ARBs) [3]. KDIGO guidelines also recommend treatment with statins for all adults ≥ 50 years with CKD, regardless of proteinuria status [3, 6].

Despite these recommendations, adherence to CKD guidelines is low; for instance, proteinuria screening rates in CKD patients range from 10 to 45 % across different health systems. [7–10] Similar deficiencies in CKD guideline adherence have been reported for achievement of optimal blood pressure goals [7–9], prescription of indicated ACEIs and ARBs [11–13], and prescription of statin therapy [14, 15]. Primary care providers manage the majority of CKD patients and often do not list CKD as a problem list diagnosis [8]. Thus, effective interventions, delivered in the primary care setting, are needed to improve screening and treatment for CKD.

Pharmacist medication therapy management (MTM) has been shown to be effective in treating hypertension [16, 17], diabetes [18], and CKD-related anemia [19]. We performed a pilot, cluster-randomized trial of outpatient primary care clinics to examine the feasibility of using pharmacist MTM to improve proteinuria screening and CKD management in a large, integrated health system.

## Methods

The pilot study was a prospective 2-arm, cluster-randomized pragmatic trial, funded by Geisinger Clinic. We recruited participants from seven primary care clinic sites at Geisinger, a large integrated health system that includes 43 community practice clinic sites across central and northeastern Pennsylvania and an extensive pharmacist-led MTM program. The Geisinger Clinic institutional review board (IRB Number 2014-0251) approved the protocol. A modified informed consent process was utilized, which entailed full disclosure and explanation to eligible patients (delivered by mail) and to participating providers (by email) at the primary care sites. Eligible patients were asked to respond within 7 days if they wished not to participate; if no response was received participation was assumed. Health care providers were informed of their patients’ participation in the study.

### Study population

Trial participants were adults actively receiving primary care with Geisinger Health Plan insurance at one of the 7 Geisinger primary care clinic sites with eGFR between 45 and 59 ml/min/1.73 m^2^ and uncontrolled blood pressure (mean blood pressure over past year ≥ 150/85). To ensure that patients were regularly receiving care at Geisinger, we only included those who had at least two outpatient blood pressure measurements within the last 12 months, and a serum creatinine scheduled within the next 30 days. Exclusion criteria included hospitalization in the past year or positive pregnancy test during the past 12 months. The first participants were enrolled into the study in September 2014 and the last participant finished the study in February 2016.

### Participant flow

We utilized a cluster randomized design, with primary care sites at Geisinger defined as the cluster unit. From a total of 43 primary care clinic sites, an initial six sites were selected based on the presence of an MTM pharmacist on site and similar numbers of patients, physicians and physician assistants. These six initial clinic sites were randomized 1:1 using a computer program into the pharmacist MTM or a control arm. A 7th clinic site was added in December 2015 due to slow recruitment in the control arm. We used the electronic health record (EHR) to identify eligible patients.

### Pharmacist MTM and control arms

All Geisinger clinics include a pharmacist trained in the management of common chronic conditions, such as dyslipidemia, diabetes, and hypertension. Training for hypertension management included a lecture from a hypertension expert, 8 week self-study of hypertension training modules, clinician shadowing, and an annual hypertension competency exam. The approach to hypertension management included assessing medication adherence, lifestyle modification (Dietary Approaches to Stop Hypertension dietary pattern and weight loss for those overweight/obese, exercise >30 min 5×/week), medication initiation/titration according to Journal of the American Medical Association (JAMA) Hypertension 2014 guidelines [20], and appropriate dosing of medications according to renal function. Under a collaborative practice agreement between pharmacists and providers, pharmacists were authorized to prescribe and titrate antihypertensive medications.

Patients in the control clinics did not receive any additional care for this study although all primary care providers received a lecture and brief written summaries about KDIGO guideline-based CKD risk stratification and management before the study began [3]. Patients in the clinics randomized to the pharmacist MTM arm received additional support from the pharmacist at the clinic site. These pharmacists received additional education about KDIGO-based screening and management guidelines for proteinuria, blood pressure and lipids in a one-on-one session with a nephrologist, who maintained communication with pharmacists regularly to answer any questions and discuss management. These pharmacists received a list of the study participants and were instructed to review charts, order lipid and urine albumin/creatinine ratio (ACR) screening tests, and manage blood pressure and lipid therapy according to KDIGO guidelines. In general, patients were contacted by telephone if they needed proteinuria screening completed, and if needed, were scheduled for clinic visits with the pharmacist for medication initiation and/or titration. If patients had completed proteinuria screening within the past 12 months and the most recent outpatient blood pressure reading in the EHR was within target range, no contact was made with the patient. Selection and titration of antihypertensive medications was based on JAMA 2014 Hypertension Guidelines [20], but to KDIGO 2012 target blood pressure goals [3]. Once the blood pressure goal was achieved, the patient was discharged from the pharmacists’ care.

### Outcomes

The primary study outcome was screening for proteinuria by urine ACR or protein/creatinine ratio (PCR) within 1 year of the enrollment date. Exploratory outcomes included screening for hyperlipidemia within 1 year of the enrollment date, end-of-trial statin treatment, achievement of blood pressure goals (<140/90 for non-proteinuric CKD and <130/80 for proteinuric CKD; patients with no proteinuria data were assumed to be non-proteinuric), and treatment with ACEI or ARBs for proteinuric CKD. Outcomes were ascertained by extraction of EHR data and chart review. Blood pressure outcomes were assessed by averaging all outpatient clinic blood pressure readings up to 1 year after the enrollment date. Proteinuric CKD requiring more aggressive BP goal and ACE/ARB therapy was defined as ACR ≥ 30 mg/g for patients with diabetes and ACR ≥ 300 mg/g for patients without diabetes, per KDIGO guidelines [3]. Protein/creatinine ratio values (g/g) were converted to urine albumin/creatinine ratios (mg/g) by dividing protein/creatinine ratios by 0.0017566 if female and dividing by 0.002655 if male [21].

### Questionnaires

At the end of the study, we administered telephone surveys of patients, providers, and pharmacists to assess the acceptability of the pharmacist MTM intervention. Patients were asked about the effectiveness of the pharmacist in providing kidney care, their comfort level with the pharmacist, and whether they wished to receive further pharmacist care. Pharmacists were asked about their confidence in managing blood pressure and lipids in patients with CKD, how helpful they believed their actions were for patients, and whether pharmacists should play an active role in managing blood pressure, lipids, and other aspects of CKD. Providers were asked whether they were aware that the pharmacist was managing some of their patients with CKD, and if so, how confident and satisfied they were in the pharmacists managing blood pressure and lipid-lowering medications. Providers were also asked whether pharmacists should play an active role in managing blood pressure, lipids, and other aspects of CKD.

### Other variables of interest

We used data from the clinic visit closest to the enrollment date to determine baseline characteristics including age, gender, systolic blood pressure, diastolic blood pressure, serum creatinine, smoking status, use of statin, angiotensin converting enzyme inhibitor (ACEI), and angiotensin receptor blocker (ARB) medications, and International Classification of Diseases, Ninth Revision (ICD-9) diagnoses of hypertension, diabetes, coronary artery disease, dyslipidemia, and heart failure.

### Analytic considerations

Assuming proteinuria screening rates of 30 % in the control group and 80 % in the intervention group, we estimated that a sample size of 72 patients would give us > 80 % power to detect a difference in screening rates between study arms at an alpha level of 0.05 with a small-moderate intracluster correlation coefficient.

Descriptive statistics including mean and standard deviations for continuous variables, and frequency percentages for categorical variables are presented. Demographic and clinical characteristics between the MTM and the control arms were compared using t-test and chi-squared tests for continuous and categorical data, respectively. We used random effects logistic regression to determine the effects of pharmacist MTM on each outcome in the total population. For the proteinuria screening outcome, we conducted a subgroup analysis only including patients who were unscreened at baseline, accounting for the clustered design. We also conducted sensitivity analyses, adjusting for differences in baseline age, diabetes, and adherence to the particular outcome. For the proteinuria screening outcome, this analysis only included the previously unscreened subgroup, and only adjusted for age as all patients with diabetes successfully achieved this outcome.

All analyses were performed using SAS Statistical Software (v9.4, SAS Institute, Inc. Cary, NC).

## Results

**Fig. 1:**
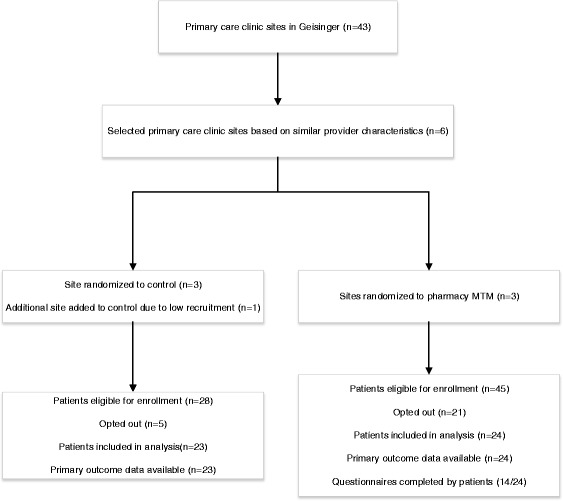
Study flow diagram

There were 73 patients eligible for the study (45 in clinics randomized to the pharmacist MTM, 28 in control clinics) who were contacted from September 2014 to February 2015. Of the 73 eligible patients, 26 patients (pharmacist MTM 21, control 5) opted out of the study prior to any intervention, leaving 24 pharmacist MTM patients and 23 control patients with data for analysis (Fig. 1). Of the 24 pharmacist MTM patients, a total of 15 (62.5 %) patients were contacted by telephone and 7 (29.2 %) also had in-person visits with a pharmacist for the trial. Among those who received telephone calls, the median [interquartile range (IQR)] number of telephone calls was 1 (1, 3). Among those who had in-person visits, the median (IQR) number of visits was 2 (1, 9). Midway through the trial, the principal investigator left the institution, and an interim analysis was conducted to determine whether to continue recruitment. After review of preliminary data, the remaining study team did not believe additional information would be gained from this pilot study, and recruitment was stopped.

### Baseline characteristics

Patients in the control group tended to be older (control 70.6 y, intervention 64.0 y; *p* = 0.06), and more likely to have an ICD-9 diagnosis of diabetes (control 35 %, intervention 17 %; *p* = 0.2) (Table 1). Otherwise, groups were similar in terms of prevalence of ICD-9 diagnoses of hypertension (control 96 %, intervention 92 %; *p* = 0.6), dyslipidemia (control 74 %, intervention 67 %; *p* = 0.6), coronary artery disease (control 17 %, intervention 21 %; *p* = 0.8), and congestive heart failure (0 % in both groups). Mean baseline eGFR was similar in both groups (54 ml/min/1.73 m^2^; *p* = 0.9), as was systolic blood pressure (control 142 mmHg, intervention 145 mmHg; *p* = 0.6), diastolic blood pressure (control 78 mmHg, intervention 81 mmHg; *p* = 0.4), use of ACEI or ARB medications (control 78 %, intervention 71 %; *p* = 0.6), and use of statin medications (control 70 %, intervention 54 %; *p* = 0.3). At baseline, 24/47 (50.1 %) of the patients already met the proteinuria screening goal, 19/47 (40.4 % already met blood pressure goals, and 44/47 (93.6 %) already met lipid screening goals.

**Table 1:** Baseline characteristics

	Control (*n* = 23)	Pharmacist MTM (*N* = 24)
Age, y	70.6 (9.7)	64.0 (13.2)
Female, %	52.2 %	62.5 %
SBP, mmHg	142.3 (16.4)	145.1 (19.7)
DBP, mmHg	77.7 (11.4)	81.3 (15.1)
eGFR, ml/min/1.73 m^2^	53.8 (5.8)	54.1 (7.7)
Current smoker, %	4.3 %	8.3 %
Hypertension, %	95.6 %	91.7 %
Diabetes, %	34.8 %	16.7 %
Coronary artery disease, %	17.4 %	20.8 %
Dyslipidemia, %	73.9 %	66.7 %
Heart Failure, %	0 %	0 %
Taking ACEI/ARB, %	78.3 %	70.8 %
Taking statin, %	69.6 %	54.2 %
ACR category^a^, %		
<30 mg/g	47.8 %	62.5 %
30–299 mg/g	26.1 %	20.8 %
> = 300 mg/g	4.4 %	4.2 %
Missing	21.7 %	12.5 %

^a^ACR category reflects urine ACR and protein/creatinine ratio values measured during baseline or during the trial for individuals missing baseline data (*n* = 23). Protein/creatinine ratio values (g/g) were converted to urine albumin/creatinine ratios (mg/g) by dividing protein/creatinine ratios by 0.0017566 if female and dividing by 0.002655 if male

Abbreviations: *SBP* systolic blood pressure, *DBP* diastolic blood pressure, eGFR estimated glomerular filtration rate, *ACEI* angiotensin converting enzyme inhibitor, *ARB* angiotensin receptor blocker, *ACR* albumin/creatinine ratio

### Effect of pharmacist MTM on proteinuria and lipid screening

At the baseline visit, 10/24 (41.7 %) of the pharmacist MTM and 14/23 (60.9 %) in the control group had already achieved the proteinuria screening goal (*p* = 0.2). At the end of the trial, proteinuria screening was not significantly different between the pharmacist MTM group and the control group (87.5 % vs. 73.9 %; OR 2.6, 95 % CI: 0.47–14.0; *p* = 0.3). However, among patients who were previously unscreened, 11/14 (78.6 %) in the pharmacist MTM group and 3/9 (33.3 %) in the control group completed proteinuria screening (OR 7.3, 95 % CI: 0.96–56.3; *p* = 0.05) (Table 2). Findings in the previously unscreened subgroup remained consistent after adjustment for age, (OR 15.0, 95 % CI: 1.1–201.7; *p* = 0.04). The proportion of patients who completed lipid screening increased from 87.5 to 100 % in the pharmacist MTM group; all patients in the control group had already completed lipid screening at baseline.

**Table 2:** Study outcomes pre- and post-intervention

	Control (*n* = 23)		Pharmacist MTM (*n* = 24)			
	Baseline	End-of-trial	Baseline	End-of-trial	OR (95 % CI)	*P* value
Proteinuria screening^a^	14 (60.9 %)	17 (73.9 %)	10 (41.7 %)	21 (87.5 %)	Entire population: 2.6 (0.5–14.0) Previously unscreened subgroup:7.3 (0.96–56.3)	Entire population: 0.3 Previously unscreened subgroup: 0.05
Lipid screening	23 (100 %)	23 (100 %)	21 (87.5 %)	24 (100 %)	N/A	N/A
Treatment with statin	16 (69.6 %)	17 (73.9 %)	13 (54.2 %)	12 (50.0 %)	0.4 (0.1–1.3)	0.1
Achieved BP goal	9 (39.1 %)	13 (56.5 %)	10 (41.7 %)	13 (54.2 %)	0.9 (0.3–3.0)	0.9

^a^Random effects logistic regression was used to determine the effects of pharmacist MTM on proteinuria screening in the entire population, and then just among patients who were unscreened at baseline, accounting for the clustered design

### Effect of pharmacist MTM on blood pressure and lipid management

Pharmacist MTM had no significant effect on blood pressure control or statin therapy (Table 2). The proportion of patients in the pharmacist MTM group who achieved their blood pressure goal was 42 % at baseline and 54 % at the end of the trial compared to 39 % at baseline and 57 % at the end of the trial in the control group (OR 0.9, 95 % CI: 0.2–3.0, *p* = 0.9). Findings were consistent when adjusted for differences in baseline characteristics (OR 0.7, 95 % CI: 0.2–2.6; *p* = 0.5). The pharmacist MTM intervention also had no significant effect on statin therapy (OR 0.4, 95 % CI: 0.1–1.3; *p* = 0.1); the proportion of patients in the pharmacist MTM group who were on statins was 54 % at baseline and 50 % at the end of the trial compared to 70 % at baseline and 74 % at the end of the trial in the control group. Results were largely unchanged after adjustment for baseline characteristics (OR 0.2, 95 % CI: 0.02–2.4; *p* = 0.2). The proportion of proteinuric CKD patients taking an ACEI or ARB increased from 2/4 (50 %) at baseline to 4/4 (100 %) at the end of the trial in the pharmacist MTM group; 3/3 (100 %) patients in the control group were taking an ACEI or ARB at the beginning and the end of the trial. Orders written by pharmacists in the pharmacist MTM arm included urine albumin/creatinine ratios (14), lipid profiles (1), statin medications (5), ACEI or ARB medications (3), and other blood pressure medications (2).

### Acceptability of pharmacist MTM

A total of 14/24 (58.3 %) of patients, 9/9 (100 %) of primary care providers, and 4/4 (100 %) pharmacists of the three intervention clinic sites answered end-of-study surveys. Only 6/14 (43 %) patients recalled having contact with a pharmacist during the trial; these patients in general were very satisfied with the care they received, felt better-informed about kidney health, and were interested in receiving future pharmacist care (Table 3). All four pharmacists felt at least somewhat comfortable with managing blood pressure and lipids although one of the 4 pharmacists did not feel that adequate CKD training was provided (Table 4). All pharmacists agreed that pharmacists could play an important role in helping manage blood pressure, lipids, and other aspects of CKD. Of the nine primary care providers, only 5 (56 %) reported being aware that their clinic site pharmacist was actively managing some of their CKD patients. There was some variability on whether providers agreed with changes made by the pharmacist during the study (2 strongly agreeing, 1 somewhat agreeing, 3 neutral, 1 somewhat disagreeing, 2 no response) (Table 5). However, most providers reported feeling comfortable with pharmacists managing blood pressure and lipids in their patients, and thought that pharmacists could be helpful in managing other aspects of CKD.

**Table 3:** Patient survey

Question	Score (SD)^a^
I was comfortable receiving care from a Pharmaciset to help manage my blood pressure, lipids, and kidney health	1 (0)
I trusted the Pharmacist and followed his/her instructions as I would follow from the doctor	1.2 (0.4)
The Pharmacist was able to answer all of my questions about the management of my blood pressure, lipids, and kidney health	1.2 (0.4)
I felt more informed about kidney health after meeting with the Pharmacist	1.8 (1.6)
I felt that receiving care from a Pharmacist was beneficial to my health	1 (0)
I would like to receive more Pharmacist attention in addition to my routine care	1.2 (0.4)

A total of 14/24 (58.3 %) patients in the intervention arm completed the survey although only six patients remembered talking with the pharmacist. All 6 of these patients completed all survey questions

^a^Score – ranged from 1 to 5 (1 strongly agree, 2 somewhat agree, 3 neutral, 4 somewhat disagree, 5 strongly disagree)

**Table 4:** Pharmacist survey

Question	Score (SD)^a^
I was comfortable providing care to patients with CKD	2.3 (1.3)
I felt that I received adequate training in order to effectively manage blood pressure and lipids in patients with CKD	2.3 (1.9)
I felt comfortable in my ability to manage blood pressure and lipids in patients with CKD	1.5 (0.6)
I felt that patients were following my recommendations	2.3 (0.5)
I felt that my actions were beneficial to the patients	2 (0.8)
I think that pharmacists can play an important role in helping manage blood pressure and lipids in patients with CKD	1.3 (0.5)
In my opinion, pharmacists can be helpful in managing other aspects of CKD beyond blood pressure and lipids	1.3 (0.5)

All 4/4 of pharmacists in the pharmacist MTM arm completed all survey questions

^a^Score – ranged from 1-5 (1 strongly agree, 2 somewhat agree, 3 neutral, 4 somewhat disagree, 5 strongly disagree)

**Table 5:** Provider survey

Question	Number (%) or Score (SD)^a^	No response
I was comfortable with the Pharmacist managing blood pressure and cholesterol-lowering medication.	1.5 (0.9)	1 (11.1 %)
I agreed with the changes that were made by the Pharmacist during this study.	2.4 (1.1)	2 (22.2 %)
I was satisfied having extra help from the Pharmacist in managing conditions in patients with CKD.	1.7 (1.0)	2 (22.2 %)
I felt that this program was beneficial to the patients with regards to CKD management.	1.9 (1.1)	2 (22.2 %)
I think that Pharmacists could be helpful in managing other aspects of CKD beyond blood pressure and lipids.	1.4 (0.7)	0

All 9/9 of providers completed the survey. However, only 5/9 (56 %) reported being aware that a pharmacist was assisting with the management of some of their CKD patients

^a^Score – ranged from 1 to 5 (1 strongly agree, 2 somewhat agree, 3 neutral, 4 somewhat disagree, 5 strongly disagree)

## Discussion

In this pilot study, we evaluated the feasibility of conducting a pharmacist-based intervention to improve adherence to KDIGO guideline-based clinical practice. One of the notable lessons was that adherence to some guidelines was already fairly good at baseline (proteinuria screening 50.1 %; achievement of blood pressure goal 40.4 %; lipid screening 93.6 %; use of ACEI/ARB for proteinuric CKD 71.4 %). Indeed our pharmacist intervention was effective in improving proteinuria screening only in previously unscreened patients, and we were not powered to show significant differences in blood pressure or statin treatment outcomes. Still, our pilot study provides valuable insights in conducting a pragmatic randomized trial in a health system, and suggests that pharmacist MTM to provide CKD care is feasible and generally well-accepted by patient, pharmacists, and providers.

A few studies have examined the use of non-physician providers in addition to nephrologists to improve management of CKD. A randomized trial of 474 patients with CKD in Canada found no benefit of nurse-coordinated care in collaboration with a nephrologist versus usual care, on eGFR decline or controlling risk factors [22]. Initial results from a randomized trial of 788 patients in the Netherlands found no effect of nurse practitioner support, in addition to nephrologist care, on cardiovascular risk or renal outcomes [23]. However, extended follow-up analysis of this trial found that nurse practitioner support decreased risk of a combined renal outcome (doubling of creatinine, ESRD or death) by 20 % [24]. Recently, a randomized pragmatic trial of 2199 veterans found that a telephone-based pharmacist intervention improved CKD-related lab testing and increased the number of antihypertensive medications prescribed, but had no significant effect on blood pressure [25]. However, pharmacists in this study were limited to one-time telephone contacts with patients and could only recommend medication changes to primary care providers.

Variability in the success of team-based approaches on blood pressure control may be due to differences in the intervention, study population, and access to nephrology care. Important intervention characteristics to consider include the intensity of the intervention, the ability of non-physicians to order medications, and the method by which blood pressure is measured. Recent randomized trials utilizing home blood pressure monitoring, delivered by pharmacists, have demonstrated success in achieving improvements in blood pressure control [16, 17]. Whether or not these successes can be translated to patients with CKD, who are among the most difficult to control, remains to be seen.

There were several valuable lessons learned during this pilot study that may help inform conducting future pragmatic trials in health systems. Many patients initially included in the study met their blood pressure and other KDIGO goals at their most recent appointment, and did not receive any specific pharmacist MTM intervention. However, blood pressure readings can vary substantially, and some of these controlled patients later had blood pressure readings higher than their goal range. Use of real-time registry data may assist pharmacists/providers in tracking blood pressure readings over time and intervening when appropriate. Some providers and pharmacists questioned the appropriateness of following KDIGO guidelines that conflicted with 2013 ACC/AHA lipid guidelines [26] and JAMA 2014 blood pressure guidelines [20]. We speculate that confusion over different guidelines may impact the success of implementing more stringent KDIGO recommendations for blood pressure and statin treatment in patients with CKD. Recent publication of the SPRINT trial, showing benefits of a systolic blood pressure goal < 120 mmHg in non-diabetic patients at high cardiovascular risk, will likely impact blood pressure guidelines [27]. Ongoing education and multidisciplinary discussion is needed to establish consensus target goals and optimize management of patients with CKD.

An unexpected complication of the trial was patient concern created by the ‘opt-out’ letter mailed to eligible patients during the early phase of the trial. While actively receiving care, several patients were not aware that they had CKD. The reasons for the lack of awareness cannot be identified. However, it seems likely that providers are either not recognizing or prioritizing moderate CKD, and thus may not have educated their patients about this condition. It is also important that future pragmatic trialists utilizing a similar, modified informed consent process recognize the possibility of disease unawareness amongst eligible patients. As a result of the opt-out process, there may have been selection bias as more patients in the pharmacist MTM arm opted out of the study compared to patients in the control arm. Future pragmatic trials trying to optimize CKD management should consider altering the study design (e.g. delayed randomization), such that waived consent is acceptable to the IRB, provided risk is minimal. This would help prevent selection bias that may have occurred by patients opting out of the research study. Considering concerns about lack of representativeness in past trials and the desire to demonstrate real-world effectiveness, pragmatic trials will likely become more and more common in the future [28].

There were limitations in our small pilot study. Cluster randomization of a small number of clinics resulted in some marginal differences in baseline age and diabetes status and recruitment was stopped early; however, the goal of the study was less about efficacy and more about identifying potential barriers for a full pragmatic trial. Perhaps the most important result from this pilot study was that power may be a significant concern – many patients were already adherent to KDIGO screening and management guidelines. A full pragmatic trial may require a system-wide intervention, or even involvement of multiple health systems. Utilization of other healthcare staff or automated ordering in risk stratifying CKD patients may be needed to optimize healthcare resources. Another limitation was that blood pressure was measured routinely in clinic and not standardized. A future pragmatic trial involving blood pressure as a major outcome needs to standardize clinic blood pressure readings in a way that minimizes interruptions to clinic workflow, and may want to consider utilizing home blood pressure monitoring. Although the vast majority of patients reported high levels of satisfaction with the pharmacist MTM arm, there was a higher rate of opt-out in this arm, which may have resulted in more compliant patients in the pharmacist MTM arm. Our study population was limited to a mostly white, rural population in an integrated health system; thus, results may not be generalizable to other populations. On the other hand, our pragmatic study design in an integrated health system represents a strength. The intervention was easily implementable given the infrastructure at Geisinger where pharmacists are available at all primary care clinic sites and already actively managing diabetes, anticoagulation, and hypertension. Both pharmacists and providers expressed considerable enthusiasm for extending management to include more aspects of CKD care. Future pragmatic trials involving pharmacists may want to incorporate other dimensions of CKD care, such as CKD-related drug safety.

## Conclusion

In conclusion, pharmacist MTM improved proteinuria screening in previously unscreened patients with CKD, and was generally well-received by patients, pharmacists, and providers. Future research is needed to examine whether pharmacist MTM could improve KDIGO guideline adherence and CKD patient outcomes.

## Abbreviations

ACEI: Angiotensin convertin enzyme inhibitor
ACR: Albumin/creatinine ratio
ARB: Angiotensin receptor blocker
CKD: Chronic kidney disease
eGFR: estimated glomerular filtration rate
EHR: Electronic health record
ICD-9: International Classification of Diseases, Ninth Revision
KDIGO: Kidney disease improving global outcomes
MTM: Medication therapy management
PCR: Protein/creatinine ratio
